# Microwave Imaging of Cotton Bales

**DOI:** 10.3390/s8117241

**Published:** 2008-11-14

**Authors:** Mathew G. Pelletier, Edward M. Barnes

**Affiliations:** 1 United States Department of Agriculture, Agricultural Research Services, Cotton Production and Processing Research Unit, Lubbock, TX. USA; 2 Cotton Incorporated, Director Agricultural Research, Cary, NC, USA; E-mail: ebarnes@cottoninc.com

**Keywords:** Cotton, Moisture, Sensing, Tomography, Inverse Solution, Image processing, Machine vision

## Abstract

Modern moisture restoration systems are increasingly capable of adding water to cotton bales. However, research has identified large variations in internal moisture within bales that are not readily monitored by current systems. While microwave moisture sensing systems can measure average bale moisture, this can be deceptive where water is unevenly distributed. In some cases, localized internal moisture levels exceed 7.5%, the upper safe limit for cotton bale storage, as determined by the USDA, as above this level, bales degrade and lose value. A high proportion of stored bales containing excess moisture have been discovered throughout the US in increasing numbers over the past several seasons, making the detection and prevention of this occurrence a critical goal. Previous research by the authors resulted in the development of microwave moisture-sensing technology. The current study examines an extension to this technology to allow for detailed cotton bale moisture imaging. The new technique incorporates a narrow beam imaging antenna coupled to a tomographic imaging algorithm. The imaging technique was able to resolve small (< 1 cm) high-permittivity structures against a low permittivity background. Moreover, the system was able to identify structures of known permittivity with high accuracy (coefficient of determination (r^2^) > 0.99). In preliminary testing on a wet commercial UD bale, the technique was able to accurately image and resolve the location of the pre-placed internal wet layer.

## 1. Introduction

The final stage in cotton processing is cotton bale packaging. In commercial cotton bale manufacturing, cotton lint is packaged into cotton bales for transportation and these are highly compressed to minimize transportation costs. The pressing operation takes the cotton lint and then presses the lint into an industry standard uniform density “UD” bale, with the goal to provide as uniform and dense a product as possible, without interior voids for later use in the textile mill's automated spinning systems. The cleaning equipment in the cotton gins are specifically designed to remove the seeds, sticks, stems, hulls and any other large impurities before packaging the lint into the UD bale and thereby limiting non-lint contaminants to typically less than 1%. The act of packaging the lint however, entails the use of very large packing forces that are typically reduced through the use of additive moisture that is added just before the packaging operation in an effort to reduce the packaging forces and relieve stress on the equipment as moist cotton requires significantly lower packing forces to form a bale. Besides this normal operational motivation, there is also an economic incentive to add moisture as cotton is sold on a wet basis. These two issues have combined in the last several years to lead to a situation where a great number of bales have been placed into the commercial market with large amounts of excess moisture that is in large undetectable by inspection from the surface of the bale. In storage and in transport overseas, bales that were manufactured with excessive moisture levels, tend to degrade via discoloration or in extreme cases support the growth of mold that renders the fiber unusable. Recent research has shown that even in moderation, excess bale moisture causes fiber degradation and color-grade changes, [1-4]. This issue is sufficiently important and has become prevalent enough to have prompted the U.S. National Cotton Council to recommend that bales be limited to less than 7.5% moisture content. More significantly, in economic terms, the U.S. Mississippi Cotton Exchange has recently adopted this recommendation as the maximum acceptable moisture limit for bales that are placed into their exchange for trade. Further, limits have also recently been placed on producers, by the U.S. Farm Service Agency, that ruled that the moisture content for all bales placed into the Commodity-Credit “CC” loan program shall not exceed 7.5% *anywhere* in the bale. Thus, there are now several contractual obligations to produce bales that do not exceed this limit with real legal implications for failure to comply as a result of the above stated rulings.

**Figure 1.**
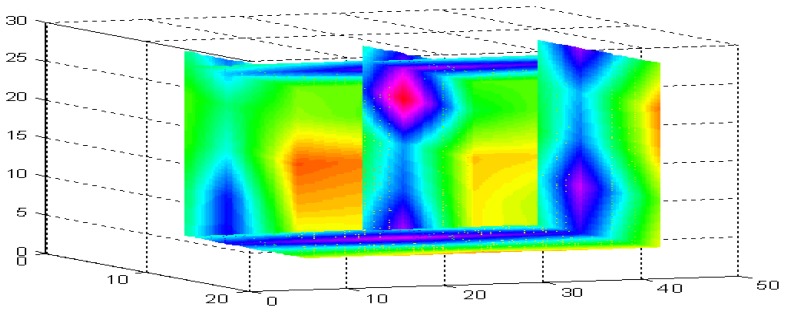
High variability of commercially produced bale, exhibiting variations in moisture from below 7% (yellow-brown) to well in excess of 12-13% (blue-pink).

Several recent studies have indicated that the moisture content within high-moisture bales are also highly variable. This variability has been shown to increase with the overall moisture content [5-7]. To illustrate the magnitude of the potential variability, a commercially produced wet UD bale was obtained, split open and then intensively sampled for gravimetric moisture. A volumetric image of the moisture distribution was constructed from approximately 100 gravimetric oven moisture samples (Figure 1) that illustrates the high variability of the internal moisture present in this commercially produced UD bale.

Given the strong economic and regulatory incentives to keep bales below 7.5% moisture, there is a clear need for systems to monitor internal moisture and moisture variability, as exhaustive moisture sampling of bales is prohibitively expensive. There are currently no systems that can detect localized interior wet spots hidden from the exterior surface. The primary objective of this research was to address this need.

## 2. Background

This work builds on the earlier success of this laboratory's microwave moisture sensors [8-11] to develop a method for imaging internal cotton bale moisture. The current system for microwave moisture measurement, [10-11], uses a very-large-aperture microwave horn to reduce multi-path interference and provide the necessary transmission gain. This large aperture effectively averages the permittivity over the entire beam area, approximately 20 × 25 cm. As this averaging precludes determination of small-scale localized moisture variability, alternative antenna designs were investigated for use in microwave imaging applications. After testing of numerous alternatives, end-fire planar antennas were found to provide a narrow beam width while maintaining a reasonable amount of antenna gain, which is required to reduce the multi-path interference common in the metal structures of cotton gins.

**Figure 2.**
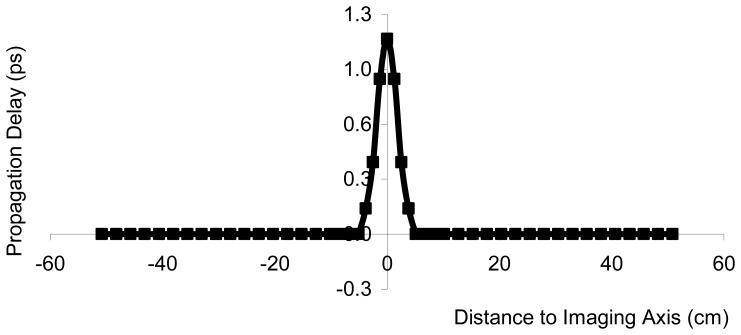
Impulse response of tomographic imaging antennas, as obtained from a scan of a single small high-permittivity object past the imaging sensors.

To examine the planar end-fire imaging antennas for use as a an imaging sensor, the proposed imaging antennas beam width was tested by passing a vial of water from left to right across the transmission line of two opposing transmit/receive pairs of the imaging antennas, while the effect upon the signal was monitored, and stored, on a microwave impedance analyzer for measurements of the through-transmission complex scattering parameters (S21). Post-analysis conversion of the scattering parameters was performed to yield propagation delay, the typical measure for bale moisture, from the complex S21 scattering parameters data. It should be noted here that cotton bales are a low loss media, therefore the measurement of apparent permittivity, provides a good estimate of real component of the complex permittivity. Results (Figure 2) suggests that sensors based upon these antennas can resolve high-permittivity objects to within 0.5-1 cm, as tested over the frequency range from 300 MHz – 3.0 GHz.

**Figure 3.**
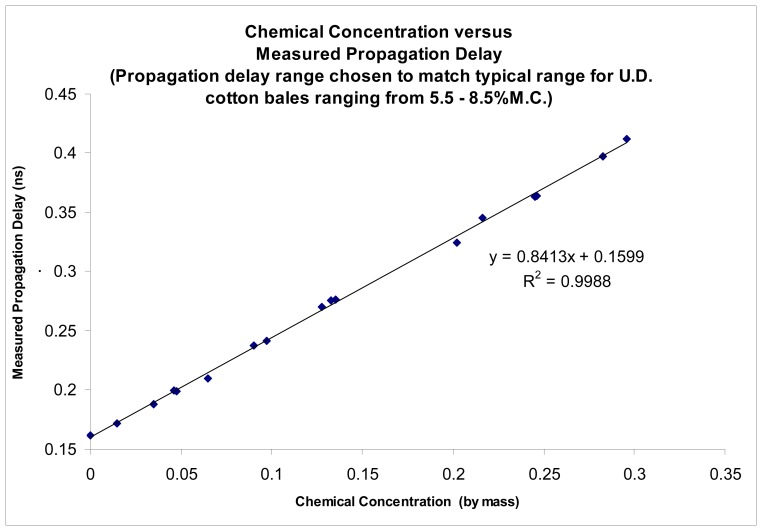
Imaging sensor's performance tested against known dielectric permittivity standards to determine accuracy of tomographic imaging antennas for straight-path permittivity sensing.

**Figure 4.**
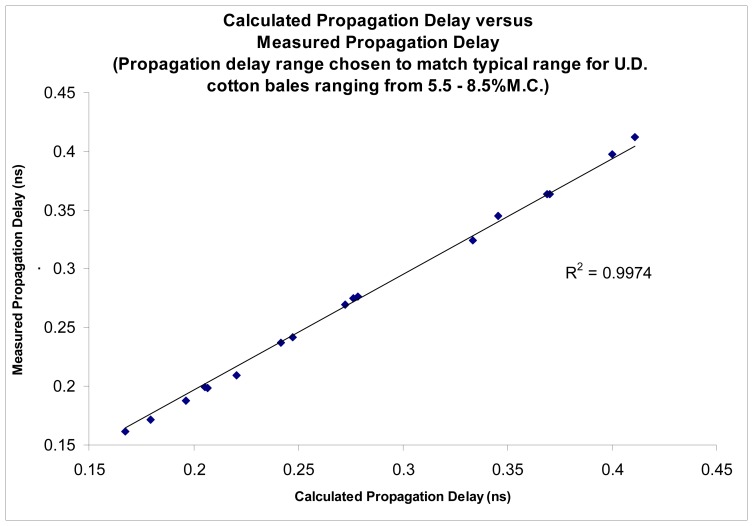
Comparison between permittivity as calculated from the known values versus imaging sensor's measured permittivity.

**Figure 5.**
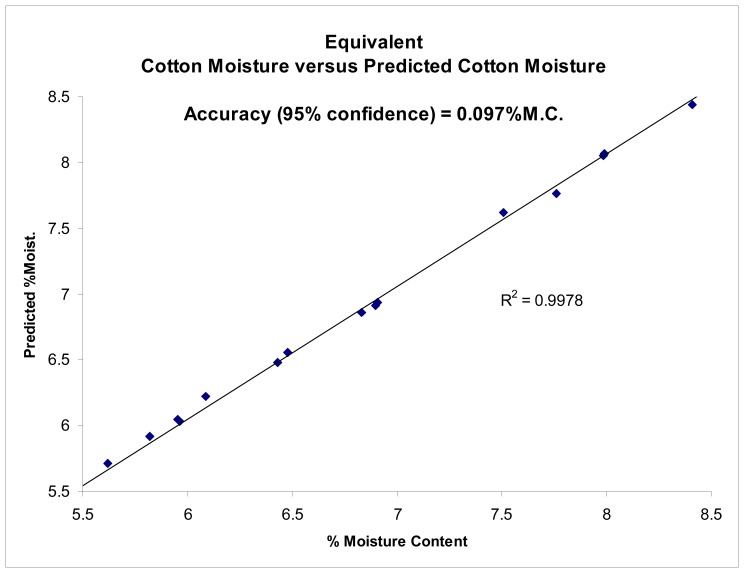
Transformation of the permittivity test of imaging sensors to equivalent permittivity of cotton bale at various moisture contents at U.D. bale density.

**Figure 6.**
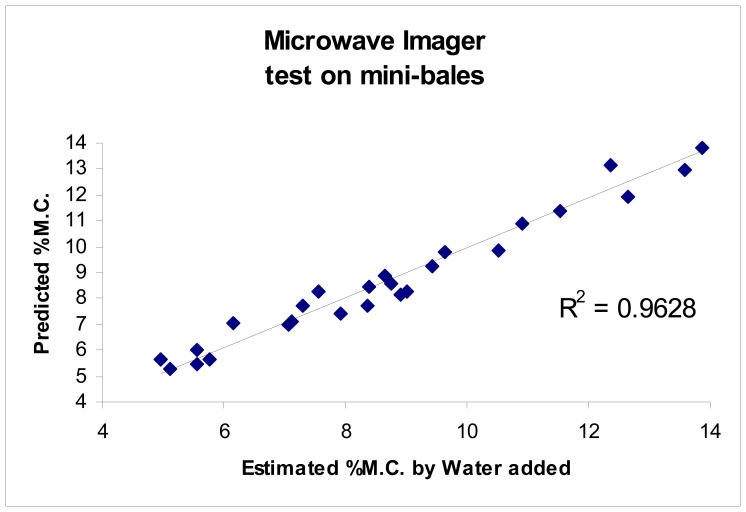
Test on mini cotton bales with imaging sensors.

The proposed sensing technique was further tested using solutions of known permittivity as well as on mini-cotton bales. The sensing technique demonstrated the ability to accurately determine permittivity of materials, as reported by Pelletier, [10]. Results are reproduced here for the convenience of the reader (Figures 3, 4, 5 and 6).

## 3. Theory and Implementation

Provided a suitable sensing element for microwave imaging, the subsequent task was to obtain a two-dimensional image from an array of the sensing elements. The first step in image reconstruction was the identification of a suitable test medium as well as a forward model to relate the measured propagation delays, obtained at each sensing element, to the internal delays of each region of the cotton bale. An important factor in determining the forward model is the strength of reflections from internal variations of permittivity. Since this strength varies for parallel and perpendicular polarity, given the polarity of the antennas, only the latter is of critical importance for this application. Note from Pozar [12], that the strength of the parallel polarized reflection coefficient “Γ” can be derived from first principles as a function of the difference in permittivities and the angle of reflection taken from the normal angle (θ = 0), as detailed in equation 1.

(1a) $$\Gamma =(\eta_{2}\cos\theta {-\eta }_{1}\cos\theta )/(\eta_{2}\cos\theta +\eta_{1}\cos\theta )$$

(1b) $$\eta_{1}=377\text{Ohms}$$

(1c) $$\eta_{2}=\eta_{1}/\text{sqr}(\varepsilon_{\text{r}})$$

where

Γ := reflection coefficient
θ := angle of reflection taken from normal angle (θ = 0)
ε_r_ := real portion of complex permittivity
η_1_ := wave impedance of air
η_2_ := wave impedance in material with permittivity of ε_r_.

From equation 1 we note that Γ is only affected by the change in permittivity. Thus, a valid test could be constructed with air as one of the dielectrics and a second corresponding to the maximum expected change in permittivity from low- to high-moisture cotton. Alternatively, low-loss dielectrics tuned to match this change could be used. One suitable low-loss stimulant would be dry and damp sand. Low moisture cotton bales have a real permittivity of approximately 2.0-2.5 and very wet bales from 3.0-4.0, yielding a change in permittivity of at most Δ(ε_r_) = 1.5. As air has a permittivity of 1.0, a suitable test stimulant material should exhibit a permittivity of 2.5. Alternatively, since air-dry sand has a permittivity of 2.9, a damp sand mixed region, simulating wet cotton, within a dry sand background, simulating dry cotton, would also provide a suitable testing platform.

**Figure 7.**
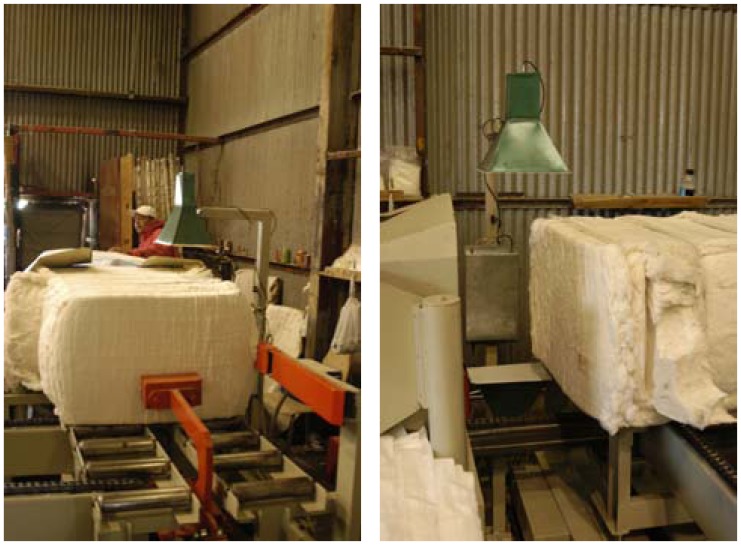
Shows how a bale is typically conveyed past the sensing station of one of the original prototype microwave moisture systems of the authors.

In seeking a commercial solution for cotton bale imaging, we note that typical cotton gins convey bales onto and off of a scale prior to bagging. Figure 7 shows the conveyance of a bale past a prototype, of one of the author's original microwave sensing systems, that measures average cotton bale moisture over a core area of approximately 20 × 25 cm^2^. The instrument is designed to take multiple readings as the bale is conveyed past the sensors, resulting in an average reading of 25 × 100 cm^2^.

**Figure 8.**
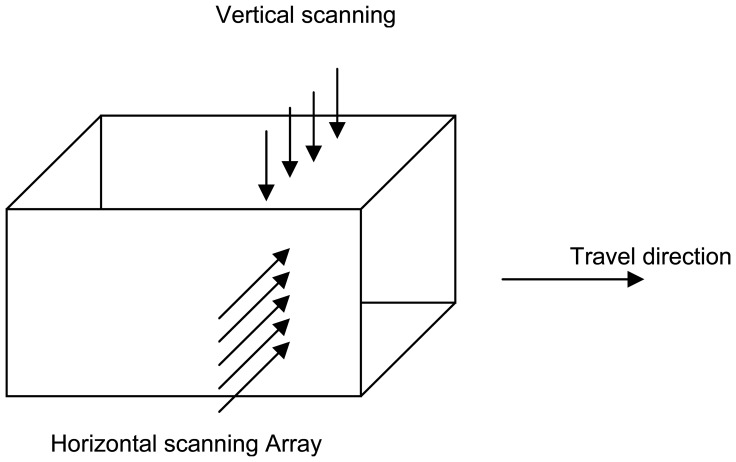
Schematic of the configuration of the scanning antennas with respect to the bale travel.

The addition of a two-dimensional array of narrow-beam permittivity imaging sensors, to the original microwave moisture system, provides an opportunity to obtain multiple cross-sectional scans during conveyance of the bale. Figure 8 shows a schematic representation of the configuration of the proposed system.

The next step in imaging is to convert the measured propagation delays from each sensor into a cross-sectional image of moisture content. As the system acquires multiple two-dimensional scans, sequential images are combined into a three-dimensional volumetric image. The physical model adopted for the two-dimensional tomographic scan assumes that microwave propagation obeys geometric optical-ray theory. This assumption is based on the high-frequency limiting case of the solution to microwave propagation via Helmholt's wave-equation [12]. The model assumes that wave energy is propagating along direct narrow paths from the source to the receiving sensor. In materials with large variations in permittivity, internal reflections would result when off-axis rays encounter large discontinuities, as previously discussed in equation 1. However, in cotton bales, the transition between low and high permittivity regions is expected to be smooth and gradual, such that internal reflections from off-axis energy are expected to be minimized, thus a first approximation of no reflection is a logical simplification of the model. The validity of this hypothesis was tested and results noted in later sections. Because the experimental permittivity system [11] measures propagation delays, the tomographic system will be constructed on the basis of time-delay measurements for each geometric ray of the forward model.

**Figure 9.**
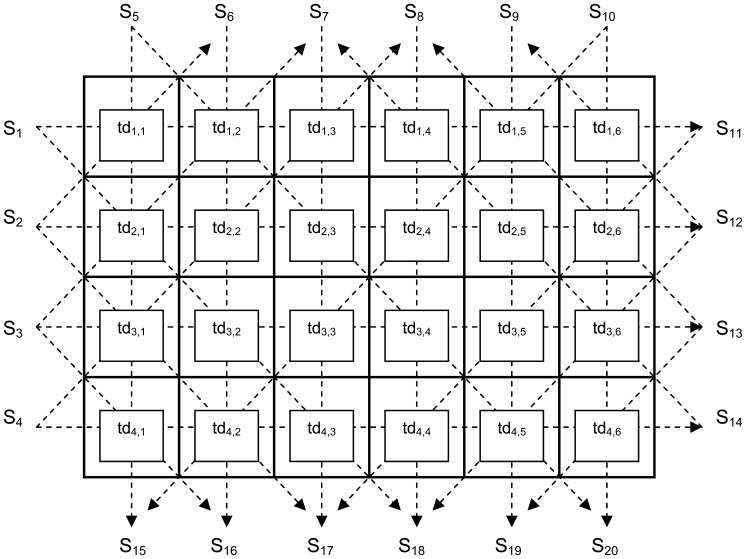
Geometric-ray model detailing the tomographic cells “(row,col)” path the signal takes during transmission from sensor S_x_ to S_y_, where S_x_ is the transmitting sensor while S_y_ is receiving sensor.

A practical system to measure internal bale moisture should provide high resolution for reasonable cost; the preliminary design proposal consists of an array of four sensors along the short dimension of the bale and six sensors along the medium dimension. This configuration permits cross-sectional scanning of the bale into 24 zones, thereby providing a high resolution image of internal moisture (Figure 8). As the bale will be scanned while it is conveyed past the two-dimensional imaging section, at an average speed of 3 seconds per bale length, the system must perform a complete two-dimensional scan in less than 25 ms if the longitudinal resolution is to be sufficient to match the proposed cross-sectional image. The corresponding tomographic geometric-ray cross-sectional model is depicted in Figure 9. Table 1 defines zone designations, with locations shown in figure 9, for the tomographic scan.

where:

sr-i := measured i-th scanned row
sc-i := measured i-th scanned column
td _Z (i,j)_ := propagation delay (per meter) for zone z_(i,j)_

Noting the microwave imaging system scans are the sum of the propagation delays across multiple zones, thus the measured path delay is the sum of all the delays in each zone that affects the scan, as for example, equations 2.

(2) $$\begin{array}{c} {\text{T}(\text{S}}_{1}➔{\text{S}}_{11})={\text{a}(\text{td}}_{\text{z}(1,1)})+{\text{a}(\text{td}}_{\text{z}(1,2)})+{\text{a}(\text{td}}_{\text{z}(1,3)})+{\text{a}(\text{td}}_{\text{z}(1,4)})+{\text{a}(\text{td}}_{\text{z}(1,5)})+{\text{a}(\text{td}}_{\text{z}(1,6)})\\ {\text{T}(\text{S}}_{1}➔{\text{S}}_{15})={\text{b}(\text{td}}_{\text{z}(1,1)})+{\text{b}(\text{td}}_{\text{z}(2,1)})+{\text{b}(\text{td}}_{\text{z}(3,1)})+{\text{b}(\text{td}}_{\text{z}(4,1)}) \end{array}$$

where:

T(S_n_ ➔ S_m_) : = total time delay along direct path from sensor n to sensor m
td_z (i,j)_ := time delay (per meter) through zone (i, j)
a := path length (m) of each cell in the horizontal direction
b := path length (m) of each cell in the vertical direction

For solving the inverse solution for the individual zones from the measured scans, the zones are configured into a continuous vector of path delays (all matrices and vectors denoted in bold):

(3) $${\text{t}}_{\text{z}}=\left\{{\text{td}}_{\text{z}(1,1)},{\text{td}}_{\text{z}(1,2)},{\text{td}}_{\text{z}(1,3)},{\text{td}}_{\text{z}(1,4)},{\text{td}}_{\text{z}(2,1)},\ldots ,{\text{td}}_{\text{z}(2,4)},\ldots ,\ldots ,{\text{td}}_{\text{z}(4,1)},\ldots ,{\text{td}}_{\text{z}(2,6)}\right\}$$

Setting up a constant matrix **A** of propagation distances through the cell along path (S_m_➔S_n_) to link the measured propagation delays “**T**” to the zone's normalized propagation delay, vector **t_z_**, yields a set of linear equations as shown in equation 4.

(4) $$\text{T}=\text{A}*{\text{t}}_{\text{z}}$$

where:

**T** : = Matrix of total time delays, comprised of all the elements “T(S_n_ : S_m_)”
**A** : = Matrix of propagation distances, comprised of path length elements
**t_z_** : = Matrix of normalize propagation delays, comprised of elements “td_z(i,j)_”
T(Sn ➔ Sm) : = total time delay along direct path from sensor n to sensor m
td_z(i,j)_ := time delay (per meter) through zone (i, j)

Noting that if only horizontal and vertical scans are measured, the system has 24 unknowns with only 10 equations, thus the system is significantly under-determined. Thus, for a practical system, the system will also need to measure numerous diagonal signal paths in order to gain enough equations to build a valid solution. We note here however the constraint of a real system, where the position of the sensors must be external to the bale, imposes some additional constraints on exploitable diagonal paths. Given these constraints along with the economic need to deploy as small a number of sensors as possible, ideally the diagonal paths should be taken to run between the existing sensors that must be placed externally to the bale. A particularly favorable choice of diagonals is detailed in figure 9, with example equations listed below in equation 5.

(5) $$\begin{array}{c} {\text{T}(\text{S}}_{2}➔{\text{S}}_{6})={\text{c}(\text{td}}_{\text{z}(1,1)})\\ {\text{T}(\text{S}}_{3}➔{\text{S}}_{7})={\text{c}(\text{td}}_{\text{z}(2,1)})+{\text{c}(\text{td}}_{\text{z}(1,2)})\\ {\text{T}(\text{S}}_{4}➔{\text{S}}_{8})={\text{c}(\text{td}}_{\text{z}(3,1)})+{\text{c}(\text{td}}_{\text{z}(2,2)})+{\text{c}(\text{td}}_{\text{z}(1,3)}) \end{array}(5)$$

where:

T(S_n_ ➔ S_m_) : = total time delay along direct path from sensor n to sensor m
td_z(i,j)_ := time delay (per meter) through zone z_(i, j)_
a := path length (m) of each cell in the horizontal direction
b := path length (m) of each cell in the vertical direction
c := path length (m) of each cell in the diagonal direction
c = √[ a^2^ + b^2^]

**Figure 10.**
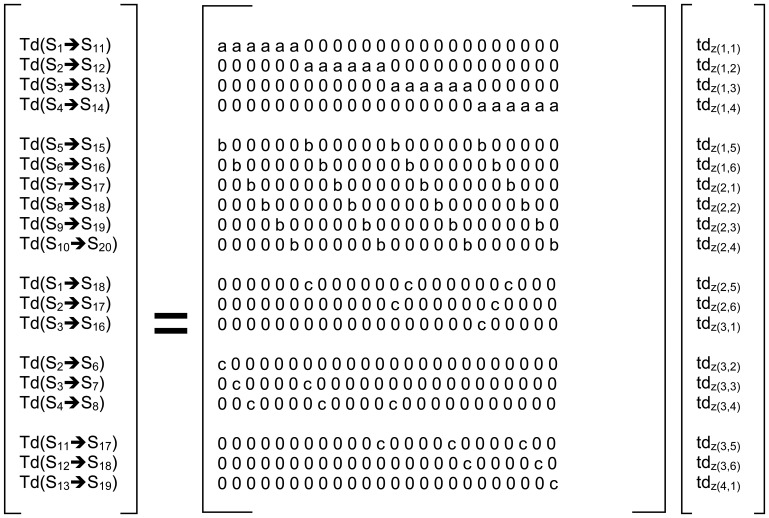
Matrix formulation for the propagation delay “**T**” for straight-path (Sx➔Sy) for the geometric-ray model; where “a”=horizontal distance through each cell, “b”=vertical distance through each cell and “c” = diagonal distance through each cell (c = √ [a^2+b^2]).

The full matrix version of the entire solution set is shown in Figure 10.

At this point it is instructive to note that even with the extra diagonal equations added to the system definition, via constant matrix **A** (Figure 10), examination of the diagonal elements of the “s” matrix from the singular-value-decomposition “SVD”, of matrix A (Table 2), reveals that while the system has 24 equations and 24 unknowns, the number of unique independent equations is only 21, which can be inferred from the fact that last three elements of the diagonal(s) of the SVD are effectively equal to zero, as noted in Table 2, which shows the system is still under-determined with a matrix rank = 21. Also of note is that the condition number of the **A** matrix is in excess of 10^16^. Therefore the system cannot be solved by conventional inverse least squares solution techniques.

Noting the system is still underdetermined and ill-formed, the standard inverse least-squares method of taking the pseudo inverse, is unsuitable for finding the inverse-solution for this system as the matrix is ill-conditioned. Furthermore, because development of a small inexpensive sensor is of primary importance, a fast solution suitable for implementation with a micro-controller, is needed. After looking at numerous techniques to solve the inverse-solution, it was determined that a single-step augmentation of the standard pseudo-inverse least squares solution, using Tikhonov regularization, was sufficient to provide rapid analysis suitable for on-line processing by a microcontroller. Tikhonov regularization as well as other inverse solution techniques are detailed throughout the literature [13-14].

The final form of the solution, using Tikhonov regularization for back calculation of the inverse-solution from the scan data, is detailed in equation 6 and and illustrated in Figure 10.

(6) $${\text{t}}_{\text{z}}={\left[\left({\text{A}}^{\text{T}}\text{A}+\Phi^{\text{T}}\Phi \right){\text{A}}^{\text{T}}\right]}^{-1}\text{T}$$

Where

**T** : = Vector of measured propagation delays; elements “T(S_n_ : S_m_)”
**A** : = Matrix of propagation distances, comprised of path length elements
**t_z_** : = Vector of interior tomography cells of normalized propagation delays,
**A**^T^ := transpose of **A** matrix
**Φ** :=α **I**
α := scalar
**I** := identity matrix

## 4. Computational time requirements for implementation of tomography algorithm on a microcontroller

As the goal of this development is to generate a low cost system for determination of moisture content of cotton bales that is augmented with information as to the distribution of the moisture, of critical need, is a solution that can be implemented in low cost hardware, preferably a small low cost microcontroller. To determine the computational requirements of the proposed solution, demanded by the previously outlined tomographic solution, equation 6, we note that **A**^-1^ = [(**A**^T^**A** + **Φ**^T^**Φ**) **A**^T^]^-1^ is a 24 × 24 element matrix that can be precomputed and stored. Thus, the solution will only require a multiply of the 24x24 element **A**^-1^ against the **T** 24 × 1 element vector. Thus, the determination of the solution, equation 6, for each slice of the cotton bale, will require 24 multiply and accumulates for each element of the final solution vector **t_z_**, which brings the total number of multiply and accumulations to 24^2^ for each tomographic slice. As most microcontrollers don't have a floating point processor on them, either floating point software routines will be required, or the algorithm will have to utilize fix-point routines, in which case the algorithm will have to undergo further analysis for determination of round-off stability for implementation in fixed-point arithmetic. As the most accurate solution will be achieved through the utilization of double precision calculations, the first test was conducted to determine the time required to achieve full double-precision accuracy. A test of the matrix-vector multiply operation was conducted on a Microchip 32 bit microcontroller running at 75MHz. The microcontroller chosen for the test was one of the fastest available that also included an on-board fixed-point multiply-and-accumulate “MAC” single-clock execution core to help minimize execution time for advanced mathematical computations. The matrix multiplication algorithm was written in the high level language C and compiled using a standard GNU compiler utilizing a floating point library optimized for this microcontroller, by Microchip, to produce standard IEEE double precision floating point arithmetic. The time requirements to perform the full 576 MACs with full double precision accuracy of the tomography algorithm for equation 6, was determined to be 2.13 ms. Thus, we find the proposed solution, as implemented on the desired hardware in full precision, is well within the time budget as estimated in the earlier section to be no more than 25 ms.

## 5. Methods

To test the effectiveness of the Tikhonov regularization inverse solution technique, simulations were conducted in order to explore the ability of the proposed solution to provide an accurate image. The simulations were designed to find the suitability of the method to find spike moisture in the two-dimensional cross-section. Given it is unlikely for cotton bales, subjected to moisture restoration systems, to exhibit true spike deviations in moisture, all simulations were conducted by constructing a true spike followed by a low pass filter. The low pass filter was designed to provide a realistic test subject for the proposed inverse solution technique.

As the forward model for the microwave tomography system utilizes a straight geometric-ray path assumption, a test of this assumption was conducted to investigate the validity of the forward model. As the geometric-ray path model ignores reflections, the necessary hypothesis for model validity requires that the microwave energy will not reflect or will minimally reflect only very low levels of energy off of any internal high-moisture areas. As the physics, discussed early in the theory section, dictates a reflection can only occur when transitioning from a low permittivity (low moisture) area to a high permittivity region, a test was designed to test for the presence of strong reflections off of internal wet bale regions.

The internal high-permittivity reflection test was conducted by taking a standard universal density UD cotton bale and breaking the bale ties to allow removal of the top layers, approximately 40 cm, off of the top of bale. A subset of the removed layers, approximately 15 cm, was sprayed with a significant amount of water to bring the lint to above 11-12% moisture. This wet layer was then returned back into its former position in the bale, which was then followed by the remaining dry cotton layers. In this manner a dry cotton bale of low permittivity was artificially created with a very wet internal layer. The goal of the creation of the wet layer bale, was to maximize the likely-hood of internal reflections by presenting a worst case scenario to the system. This was accomplished by ensuring the test bale achieved the maximum permittivity differences, as seen in normal commercial settings, occurred between the dry sections of the bale to the wet layer. The moisture content of this wet-layer was designed to be at the upper end of the expected range of ultra-wet cotton bales, as this highest level of moisture, against the very dry bale background, ensures the test presents the strongest possible internal reflector of microwave energy to the system, as noted in equation 1 from the previous discussion in the theory section.

**Figure 11.**
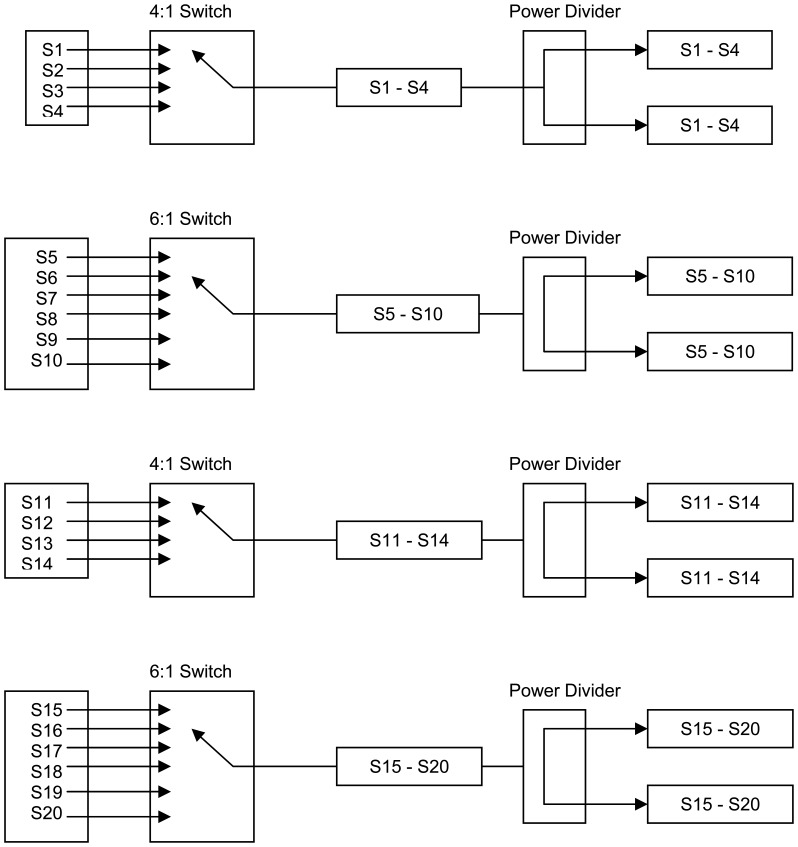
First stage switching configuration, detailing how to obtain switching between all transmit and receive signal paths required for solution of equation 6 and Figure 10.

**Figure 12.**
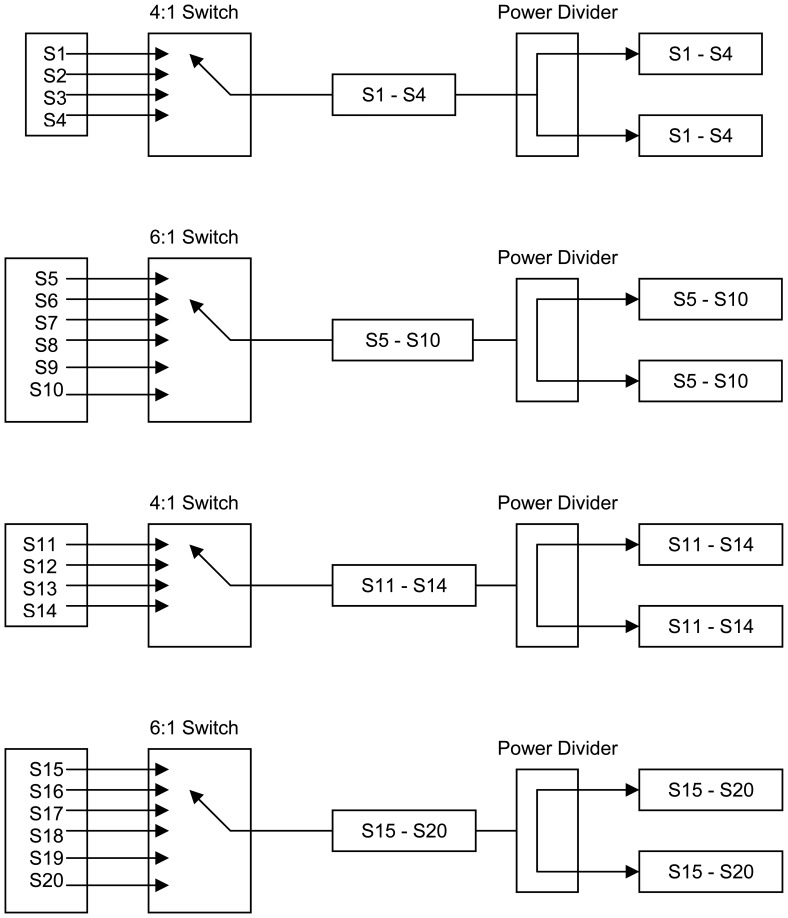
Second stage switching configuration; showing how the transmit signal propagates out either the upper bank or lower bank of 2nd-stage switching, depending upon the position of the transfer switch and the corresponding 4:1 switch (2^nd^ stage) to the 1^st^-stage 4:1 switch to the chosen transmitting antenna. In parallel, the other bank of 2^nd^-stage switching reroutes the receiveing signal, as selected by the 4:1 1^st^ and 2^nd^ stage switches. In this manner, the transmit signal is routed to any one of the antennas, which is then routed back after reception at another selected receiving antenna that is then routed to either Signal-A or Signal-B for comparison to the internal reference copy of the transmitting signal for measurement of the S21 complex scattering parameters for determination of the complex permittivity of the material.

The system was tested by transmitting, via the imaging antennas, energy through low permittivity regions, alongside the wet layers that were directly adjacent to high permittivity areas. Thus, the microwave transmission beam direction was parallel to the wet layer which would ensure the optimal geometry for reflections to occur. In the course of the test, the entire bale was scanned in this manner to allow for the development of a two-dimensional permittivity map of bale moisture. Further, as the design of the experiment ensured the presence of reflections were maximized well beyond what is expected in normal use, if reflections were of the magnitude to cause measurement errors, then the test of the system with reflections would manifest in the data by showing up with an intermediate permittivity reading for the transmission of the beam through cells adjacent to the wet layer. The next step in the study was to examine the complexity of the required switching hardware necessary to obtain all the scan-lines required by the solution of equation 6 and Figures 9 and 10. In mapping out a switching solution, one possible solution was developed, table 3 (details of circuit topology shown in Figures 11 and 12):

As Table 3 and Figures 11 and 12 indicate, a significant number of switches are required to achieve all the different sensing beams. And as off the shelf microwave switches typically range from at $100 to $1,000 per switch, a reasonably priced system will have to utilize a custom designed switching subsystem.

## 6. Results and Conclusions

In response to the increasing application of excess moisture to cotton bales by the industry's new moisture restoration systems, this study investigated the potential for utilizing low frequency microwave permittivity sensing for use in imaging of the localized variations of cotton bale moisture. As the forward model for the microwave tomography system utilized a straight geometric-ray path assumption, a test of this assumption was conducted to investigate the validity of the forward model. As the geometric-ray path model ignores reflections, the hypothesis was the expectation that the microwave energy would not reflect or would only present very low levels of reflections off of any internal high-moisture areas. To test this hypothesis, the test was designed to transmit energy through low permittivity regions that were adjacent to high permittivity areas. If reflections were a problem, then the system should have registered an intermediate permittivity reading even though the transmission was through a low permittivity reading volume portion of the dry cotton bale.

**Figure 13.**
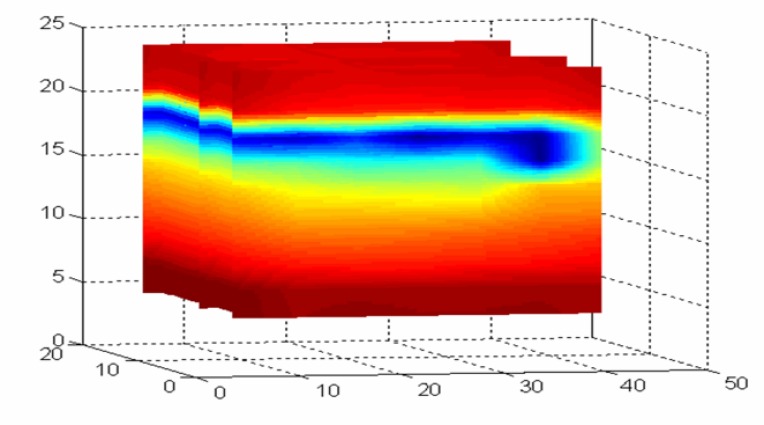
Test scan with imaging sensors of commercial UD cotton bale that was built with a 15 cm horizontal wet layer.

Utilizing multiple scans of the bale over a grid of positions across one the side of the bale with the receiving sensors directly opposing the transmitting antennas located on the opposite side of the bale, an image was constructed of the wet-layer-bale from numerous two-dimensional scans. Qualitatively it can be seen the built up tomographic image accurately displays the placement of the wet layer where it was placed within the bale, Figure 13.

**Figure 14.**
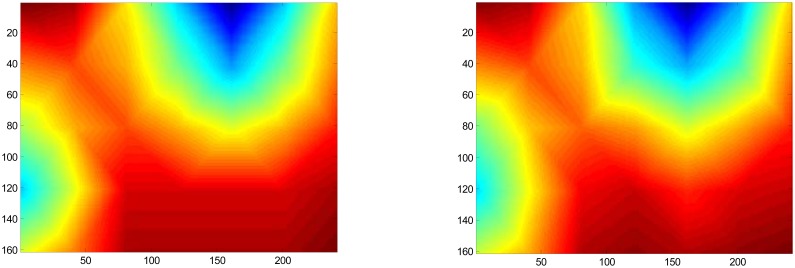
Details results from simulation of the inverse-solution method where the image holds the true Z-domain data with the solved inverse-solution to the true normalize cell propagation delays “**t_z_**”, via equation 6, depicted in the image on the right.

In examining Figure 13, to top transition from dry to wet exhibits a sharp transition, thereby suggest that internal multi-path reflections off the wet layer, have a minimal influence on the measured permittivity reading obtained through the dry cotton located immediately adjacent to the wet layer. While there does appear to be some mixing of the wet to dry layer in moving from the wet layer down to the dry layer, toward the bottom of the page, this mixing occurred in the same direction as gravimetric drainage, thus this bled was likely due primarily from the moisture drainage from the wet layer down through the dry layer located immediately below the wet layer, rather than from internal reflections. We also note that the test presents an extreme case that hopefully will never be repeated in commercial processing plants as a bale with internal moisture this high would undergo serious fiber degradation during storage, hence the need for the moisture sensors to control moisture well below these damaging moisture levels. The evidence for minimal reflections, of the parallel through-transmission measurements, from the scans obtained immediately above the wet layer, coupled with measurements of permittivity in agreement with the rest of the dry bale, confirms the hypothesis that internal wet areas are for practical purposes, non-reflective. Therefore the direct path geometric-ray model is suitable for use as the basis for the tomographic forward model. Testing of the inverse solution using Tikhonov regularization was conducted by simulation. A typical example of simulation results, contrasting the true **t_z_** domain against the inverse-solution obtained via Equation 6, is presented in Figure 14.

Values for alpha less than 0.006, equation 6, were found to be suitable across the broad spectrum of the simulation sets of this study. While artificial low-pass filtering is observed in the simulation data as a result of the damping applied by the alpha coefficient of the Tikhonov regularization to the smallest terms of the “S” matrix from the singular-value-decomposition, as cotton moisture restoration systems are designed to add moisture uniformly, a moisture spike confined to a single tomographic cell would be highly unlikely and the inherent low-pass filtering of the solution by the Tikhonov regularization solution should follow a smooth function. Given this, the low-pass filtering is of minor concern, as the primary goal is to alert operators to the presence of large variations in internal moisture. In this respect, the Tikhonov regularization provides a valid solution

The accuracy of the permittivity measurements utilizing the new imaging sensors was also tested by means of testing on known permittivity standards, as the absolute average moisture is of primary consideration for bale moisture sensing. Tests conducted to determine the accuracy of the imagers were shown to provide the ability to accurately determine the permittivity of known permittivity standards with a coefficient of determination of r^2^>0.99, Figures 3, 4 and 5. Impulse response testing also indicated the ability of the sensors to resolve localized variations in permittivity, and in combination, both of these tests suggest the system should be able to provide an accurate determination of the relative change in permittivity, especially when compared to the over-all average bale moisture which can be determined by the system in the normal method of across bale averaging [6, 8-10].

At this point it would be premature to claim that the system can accurately determine the absolute value of the permittivity on a spot level as the density of cotton bales are known to vary across the bale width due to imperfect loading within the bale press. As the spot density will affect the apparent and measured permittivity, the accuracy will be dependant upon the assumption of a uniform density. However, for the purposes of detection of internal wet spots, the large deviation in measured permittivity due to excessive moisture will significantly outweigh any small variations in density to the non-uniform density of the cotton bales. Further, as the average bale density can be determined via the fixed size of the bale, in conjunction with the known bale weight, the obtained average permittivity coupled with the average density will provide an accurate baseline for highly repeatable measurements of bale moisture., as previously demonstrated in the earlier systems of the authors.

The timing constraints imposed by settling time and accuracy, as estimated from current instrumentation [8-11], were analyzed in light of the switching required by the tomographic solution. Experience with earlier systems suggests that a scan can be tuned accurately in less than 1-ms/scan, such that the 24-zone imaging system would require approximately 25 ms. At normal bale conveyance speeds, this translates to an approximate distance between cross-sections of 10 cm (3.9 in). Given these specifications, the total number of zones imaged for bale moisture will be over 240, which should more than suffice for determination of excessive localized internal bale moisture.

In summary, this study found the following:

- New pencil beam imaging antennas were able to accurately determine the permittivity of a range of samples of known permittivity, Figure 3;
- Pencil beam imaging antennas were able to resolve localized fine structure, Figure 2;
- A topology of the switching structure (Figures 11 and 12) that supports the inverse solution tomography model, can be realized in a physical system;
- Tests for internal reflection from a wet-bale layer against a dry background provides support for the validity of the geometric-ray model as the basis for tomographic imaging of cotton bales (Figure 13);
- Simulation studies indicated the inverse solution methodology using Tikhonov regularization provides an accurate image from obtained scan-line data (Figure 14);

Based upon the results of this study, tomographic imaging of cotton bales can be accomplished via incorporation of narrow beam imaging antennas when used in conjunction with a tomographic imaging model and the switching hardware design presented in this study. The imaging system presented in this study will allow for improvements in both day to day monitoring of moisture in cotton gins and screening of bales for excess internal bale moisture before the bales are brought into long term storage. The techniques outlined in this study provide a solution to the previously intractable problem of detection of localized interior wet bale moisture that is the primary cause of fiber degradation in storage.

## Figures and Tables

**Table 1.** Definition of tomography cell domains.

	**sc-1**	**sc-2**	**sc-3**	**sc-4**	**sc-5**	**sc-6**
sr-1	td_Z(1,1)_	td_Z(1,2)_	td_Z(1,3)_	td_Z(1,4)_	td_Z(1,5)_	td_Z(1,6)_
sr-2	td_Z(2,1)_	td_Z(2,2)_	td_Z(2,3)_	td_Z(2,4)_	td_Z(2,5)_	td_Z(2,6)_
sr-3	td_Z(3,1)_	td_Z(3,2)_	td_Z(3,3)_	td_Z(3,4)_	td_Z(3,5)_	td_Z(3,6)_
sr-4	td_Z(4,1)_	td_Z(4,2)_	td_Z(4,3)_	td_Z(4,4)_	td_Z(4,5)_	td_Z(4,6)_

**Table 2.** Diagonal elements of the singular-value-decomposition of matrix A.

S = [4.3, 3.2, 3.1, 2.9, 2.9, 2.8, 2.6, 2.6, 2.3, 2.3, 2.2, 2.1, 2.0, 1.4, 1.4, 1.4, 1.4, 1.4, 1.3. 0.4, 0.3. 0.000, 0.000, 0.000]

**Table 3.** Switch requirements to implement full 24 cell two-dimensional tomographic scans for cotton bales.

**Stage 1:**	
**Quantity**	**Description**
2	4:1 switch
2	6:1 switch
4	1×2 Power Divider
**Stage 2:**	
**Quantity**	**Description**
1	2:1 switch
2	4:1 switch
1	2:2 Transfer switch
1	1×2 Power Divider
